# Psychometric Properties of the “Quality of Life in Life-Threatening Illness—Family Carer Version” (QOLLTI-F) in Persian-Speaking Carers of COVID-19 Patients

**DOI:** 10.3389/fpsyg.2022.838074

**Published:** 2022-05-03

**Authors:** Armin Fereidouni, Abbas Ebadi, Maryam Rassouli, Seyed Morteza Hosseini, Mohsen Mollahadi, Ali Khorshidvand, Mohammad Javid, Behnam Ansari, Mohammad Saeid Rezaei, Salman Barasteh

**Affiliations:** ^1^Medicine, Quran and Hadith Research Center, Baqiyatallah University of Medical Sciences, Tehran, Iran; ^2^Perioperative Nursing, Faculty Member, Department of Operating Room Technology, School of Nursing and Midwifery, Shiraz University of Medical Sciences, Shiraz, Iran; ^3^Behavioral Sciences Research Center, Life Style Institute, Baqiyatallah University of Medical Sciences, Tehran, Iran; ^4^Nursing Faculty, Baqiyatallah University of Medical Sciences, Tehran, Iran; ^5^Cancer Research Center, Shahid Beheshti University of Medical Sciences, Tehran, Iran; ^6^Exercise Physiology Research Center, Life Style Institute, Nursing Faculty, Baqiyatallah University of Medical Sciences, Tehran, Iran; ^7^Student Research Committee, Baqiyatallah University of Medical Sciences, Tehran, Iran; ^8^Health Management Research Center, Nursing Faculty, Baqiyatallah University of Medical Sciences, Tehran, Iran

**Keywords:** quality of life, family caregiver, measurement, validity, reliability, psychometric properties, scale

## Abstract

**Background:**

Measuring family caregivers’ quality of life plays a significant role in improving the quality, efficiency, development, and provision of efficient services for patients with COVID-19. As a result, evaluating the quality of life requires the use of valid and reliable measures that are culturally appropriate. This study was conducted to determine the psychometric properties of the Persian version of the Quality of Life in Life-Threatening Illness–Family Carer Version (QOLLTI – F) in patients with COVID-19.

**Methods:**

This methodological study was carried out in 2021 at Baqiyatallah Hospital in Tehran. After gaining approval from the tool creator, the translation was carried out utilizing the forward-backward approach. Cognitive interviews with 10 family caregivers of COVID-19 patients were used to demonstrate face validity. Moreover, construct validity was identified by performing exploratory factor analysis (EFA) (*n* = 251), confirmatory factor analysis (CFA) (*n* = 200), and convergent validation using Zarit Burden Interview (ZBI) questionnaire. For scale reliability, internal consistency and stability were performed using Cronbach’s Alpha Coefficient and test-retest, respectively.

**Results:**

451 family caregivers of patients with COVID-19 were enrolled in this study. Three factors with a cumulative variance of 51.85% were extracted during EFA: (1) Caregiver’s physical-emotional status, (2) Satisfaction with the situation, and (3) Caregiver’s concerns. CFA showed that the model enjoyed a moderate to a good fit of information (RMSEA: 0.087; NFI: 0.98; CFI: 0.91; IFI: 0.91; GFI 0.89; standardized RMR: 0.070). A significant correlation was found between the Persian version of the ZBI and participants’ total scores of QOLLTI – F v3 (*r* = –0.196, *P* = 0.000). Cronbach’s Alpha Coefficient = 0.719 and ICC stability reliability = 0.71 of the questionnaire were confirmed.

**Conclusion:**

The Persian version of the QOLLTI – F v3 is a valid and reliable scale that can measure family caregivers’ quality of life during a Life-Threatening illness in patients with COVID-19. This instrument may be utilized in clinical trials and research to enhance the quality of life for family carers in Iranian society.

## Introduction

In the last 2 years, the increase in COVID-19-related deaths has become the greatest threat to human health in the current century (Plohl and Musil, 2020); By November 26, 2021, 5,174,646 million people worldwide have lost their lives. By the widespread outbreak of the SARS CoV-2 virus, World Health Organization [WHO], 2020 has declared a public health emergency worldwide (Afrashteh et al., 2020).

The high prevalence of COVID-19 has negatively affected individuals and communities’ physical, social, psychological, and mental functioning which has had significant economic, social, and health consequences (Algahtani et al., 2021). Besides, it has led to increased psychosocial and physical stress such as fear of infection and death, the spread of fake news and rumors, interference in activities of daily living, travel bans and restrictions, social isolation in terms of quarantine or other distancing measures, and occupational and financial problems (Alipour et al., 2020). In this respect, the findings of Wang et al. (2021) demonstrate that COVID-19 has moderate to severe psychological effects that might cause feelings of anxiety, depression, and fear in people. Godinic et al.’s research looks at the effects of the COVID-19-related economic crisis on people’s mental and psychological health (Godinic et al., 2020; Ganesan et al., 2021). The unique nature of COVID-19, the need to maintain public safety, social distancing, limited educational and care resources, prolonged illness duration, inadequate knowledge about providing care and being an emerging disease, along with physical problems resulting from the disease might have led to increased isolation and solitude and intensified psychological stress in these patients (Holt-Lunstad et al., 2015). Moreover, compared to other patients, caring for these patients has posed additional challenges to family caregivers (Mirzaei et al., 2020). By managing care, creating proper communication, and educating patients, family caregivers assist minimize psychological and health stresses on the country’s health system and empower people to cope with disease (Dixe et al., 2019). These caregivers are often affected by the biological and physiological damage caused by the patient care process, and they are likely to experience reduced social activities, lack of leisure time, and reduced relationships with family and friends (Magliano et al., 2005). In addition to the caring role, most family caregivers are confronted with other challenges such as job management, housekeeping, childcare, concerns about their children’s education due to school closures, new economic pressures, high medical costs (Kent et al., 2020), and protecting self and family members against virus spread, all of which affect their quality of life (Dinç and Erdoğan, 2021).

Quality of life is an important measure of a society’s health and well-being, and its assessment may lead to the identification of a broad variety of issues that influence people’s everyday lives. World Health Organization defines the quality of life as an individual’s mental perception of the impact of illness or medical condition on various domains of life, including physical, mental, social, and occupational functioning (Algahtani et al., 2021). COVID-19 pandemic has negatively affected the quality of life of all strata of society, especially family caregivers (Dinç and Erdoğan, 2021). During a pandemic, support systems for family caregivers are essential to enable them to efficiently cope with spiritual and physical problems (Dinç and Erdoğan, 2021).

Therefore, it is necessary to measure family caregivers’ quality of life using reliable tools to develop and provide the most efficient services (Axelsson et al., 2020). Quality of Life in Life-Threatening Illness–Family Carer Version was designed by Cohen et al. (2006) in English and French. Its validity and reliability were confirmed. This scale measures caregivers’ quality of life and their perception of the patient’s life-threatening and challenging condition (Cohen et al., 2006). The instrument has been translated, and its psychometric properties were investigated in various cultures, including Australian (Bradford et al., 2012), Swedish (Axelsson et al., 2020), German (Schur et al., 2014), Spanish (Arias-Rojas et al., 2021), Malaysian (Alnjadat et al., 2014), Indian (Nayak et al., 2014), and Czech (Bužngová et al., 2015). However, a Persian tool to measure family caregivers’ quality of life in Life-threatening illnesses has not been designed or psychometrically investigated. Therefore, this study aimed to translate and determine the psychometric properties of the Persian version of the Quality of Life in Life-Threatening Illness–Family Carer Version in the families of patients with COVID-19 (QOLLTI – F).

## Materials and Methods

### Study Design

The present methodological study has investigated Persian version and the psychometric properties of QOLLTI – F v3 scale.

### Study Population/Sampling

The target population was family caregivers of the patients with COVID-19 referred to Baqiyatallah Hospital. Availability sampling was used in this study, and sampling was performed in 2021 for a period of 1 month. Inclusion criteria included: at least 18 years of age, caring for a patient with confirmed COVID-19 according to a physician’s diagnosis and laboratory tests, willingness to participate in the study by signing a written consent form, literacy of reading and writing in Persian, and no cognitive or mental disorders according to the individual’s report. Family caregivers who were reluctant or unable to cooperate in completing the questionnaire or who delivered an incomplete questionnaire were excluded.

### Study Instruments

#### Demographic Information Questionnaire

A researcher-made questionnaire was used to collect demographic information, including gender, age, marital status, education level, job status, and satisfaction with monthly income.

#### Quality of Life in Life-Threatening Illness–Family Caregiver Version 3

This scale was designed by Cohen et al. (2006), and its validity and reliability were confirmed. The present scale includes 16 items and seven subscales, including Carers Own State (5 items), Relationships (2 items), Carers Outlook (3 items), Quality of Care (2 items), Patient Condition (1 item), Finances (1 item), and Environment (2 items). Scale scores range from 0 to 10. The overall score of scale is calculated based on the average score of 7 subscales. The minimum and the maximum scores are 0 and 160, respectively. The scale’s validity was confirmed *via* content and construct validity, and its reliability was obtained by calculating the internal consistency and Cronbach’s alpha coefficient of 0.857. The test stability was reported to be 0.77 for the first and second tests and 0.80 for the second and third tests using the test-rest method. ICC for seven subscales was reported to be 0.50–0.79 (Cohen et al., 2006).

#### Zarit Burden Interview

Zarit Burden Interview (ZBI) is the most widely used tool to assess the care burden of family caregivers, designed by Zarit et al. (1986) (Bédard et al., 2001; Yap, 2010). This questionnaire consists of 22 items and assesses caregivers’ individual, social, emotional, and financial dimensions. The items are rated on a 5-point Likert scale ranging from 0 = never, 1 = rarely, 2 = sometimes, 3 = often, 5 = always. Each individual’s score is between zero and 88 (Zarit et al., 1986). A higher score indicates a greater psychological burden. Scores in the range of 61 to 88 indicate severe, 31–60 moderate, and less than 30 mild care burden. The validity and reliability of this questionnaire have been investigated by Navidian et al., according to Iranian culture. The Persian version of this tool has desirable psychometric indices. Besides, the test-retest reliability coefficient of the intra-cluster correlation coefficient was 0.94 at a time interval of 2 weeks (Navidian and Bahari, 2008).

### Translation Procedure

The forward-backward translation method was used. After obtaining permission from the developer in the forward translation stage, the original English version of the QOLLTI-F was translated into Persian by two specialized English translators, according to the International Quality of Life Assessment (IQOLA) protocol (Bullinger et al., 1998). Afterward, two versions of the translated scale were reviewed in a meeting with researchers in the incorporation stage. Finally, an initial joint translation was obtained as the researchers reached a consensus. In the backward translation stage, the joint Persian translation prepared in the previous stage was translated into English by two native speakers fluent in Persian and English, and an English version was obtained. Two English versions prepared in the previous step were sent to the tool developer to be compared. The developer compared the provided questionnaire to the original version conceptually and approved it.

### Face Validity

Cognitive interviews were used to assess the qualitative face validity following the translation process. A cognitive interview is conducted to identify the source of error in the scale by focusing on respondents’ cognitive process when completing the scale (Willis, 2004). Furthermore, ten family caregivers with diverse economic, social, and education levels were interviewed. They were requested to rate the legibility, clarity, and structure of the items, ease of comprehension, item difficulty, confusing words, item classification, ease of responding, language forms, and wording. Subsequently, the modifications were applied in the Persian version of QOLLTI-F.

### Construct Validity

Exploratory factor analysis (EFA), confirmatory factor analysis (CFA), and convergent validity methods were used to assess the construct validity of this scale.

#### Exploratory Factor Analysis

Exploratory factor analysis is used to discover the underlying structure of a relatively large set of variables. The minimum sample size required for EFA is 3–10 participants per item (Kellar and Kelvin, 2013). To assess EFA, 251 family caregivers of patients with COVID-19 were enrolled in the study using the convenience sampling method. Moreover, Keiser-Meyer-Olkin (KMO) and Bartlett’s tests were performed to evaluate the sample adequacy and suitability. A value of KMO closer to one is more suitable for factor analysis; however, a score greater than 0.5 is generally acceptable, and a score greater than 0.7 is more appropriate (Ebadi et al., 2017). Bartlett’s test with a significance level below 0.05 is acceptable (Kaiser, 1974; Hutcheson and Sofroniou, 1999). Favorable results of KMO and Bartlett’s tests suggest a favorable correlation matrix for factor analysis (Mohammadbeigi et al., 2015). The association between each component and each questionnaire item is known as factor loading. The association must be suitable for a question to be retained. The minimum factor loading in this study was considered 0.3. In case that factor loading is less than 0.3, the correlation between the factor and the item is weak (Hair, 2009; Rashidi Fakari et al., 2020). For factor extraction and interpretation, the principal components analysis (PCA) and the PROMAX rotation methods were used, respectively (Samitsch, 2014).

#### Confirmatory Factor Analysis

The extracted factors were assessed using CFA following EFA (Waltz et al., 2010). Afterward, 200 family caregivers were included in the study to evaluate the CFA. CFA was performed using Partial Least Squares to assess the fit of the proposed model with the data. In general, in CFA, the correlation between latent factors and measurable variables is clear; this way, the significance, and intensity of the correlation are determined. Model fit indicators in CFA are classified into three general categories (1) Absolute fit: root mean square error of approximation (RMSEA), standardized root mean square residual (SRMR), goodness-of-fit index (GFI), and Chi-square, (2) Comparative fit: comparative fit index (CFI), incremental fit index (IFI), RFI, normal fit index (NFI), TLI, and (3) Parsimonious fit: PCFI, PNFI, AGFI, AIC.

#### Convergent Validation

The last method to assess the construct validity was Convergent validation. Respondents simultaneously completed the ZBI and the Persian version of the QOLLTI-F. To confirm this method, the correlation of QOLLTI-F with the ZBI was measured using a correlation coefficient (Krabbe, 2016).

### Reliability

To assess the reliability of the scale, two methods of determining Internal Consistency and Stability were used. Cronbach’s Alpha Coefficient was calculated to measure the internal consistency of the tool. Cronbach’s alpha between 0.8–0.7 indicates acceptable and sufficient internal consistency (Rattray and Jones, 2007). To determine the tool stability, the test-retest method with a sample size of 30 was used. The retest was performed at a time interval of 14 days, and the scores obtained in these two stages were compared using the Intra-cluster correlation index (ICC). ICC index greater than 0.80 is assumed as the desired stability (De Boer et al., 2004). The total item correlation was also examined. We assessed the association between each item and the scale’s overall score before deciding whether or not to keep the questions. Questions having a correlation of less than 0.3 were eliminated from the analysis (Stevens, 2012).

### Ethical Consideration

The permission to conduct the study was obtained from the ethics committee of Baqiyatallah University of Medical Sciences (ethics code: IR.BMSU.BAQ.REC.1400.48). After obtaining permission from the tool developer *via* email, the translation was performed. Before initiating the research, study participants were informed of the study objectives, and they were recruited after signing written informed consent. The participants were ensured of data confidentiality and the right to withdraw from the study at any stage.

### Data Analysis

SPSS software version 22 and LISREL version 8.8 were used for data analysis. In all analyses, the significance level was considered *P* < 0.05.

## Results

### Socio-Demographic

Among 451 participants, 274 (60.8%) were male, and 177 (39.2%) were female, their mean age was 41.38 ± 13.62. Table 1 shows the mean score of quality of life, based on demographic characteristics. There was a statistically significant relationship between the patient’s marital status (*P* = 0.01) and the income with his/her quality of life (*P* = 0.04). However, no statistically significant relationship was found between gender (*P* = 0.01), marital status (*P* = 0.89), level of education (*P* = 0.65), and job status (*P* = 0.83).

**TABLE 1:** Mean score of quality of life, based on demographic characteristics.

Variable	*n*	%	Mean (SD)	Statistical test	Results
**Gender**					
Male	274	60.8	96.95(23.12)	Independent *T*-test	*P* = 0.897
Female	177	39.2	95.42(2270)		*T* = 0.691
**Age**					
0–20	11	2.4	88.09(21.51)	One way ANOVA	*P* = 0.2
21–40	240	53.2	95.25(23.50)		*F* = 1.59
<*40*	200	44.3	98.13(22.26)		
**Marital status**					
Single	122	27.1	93.97(26.41)	One way ANOVA*	*P* = 0.01
Married	315	69.8	96.56(21.34)		*F* = 4.16
Divorced/widowed	14	3.1	112.42(19.76)		
** Education**					
Elementary	31	6.9	99.67(24.20)	One way ANOVA	*P* = 0.65
Diploma	148	32.8	94.70(22.69)		*F* = 0.54
Bachelor’s	187	41.5	97.16(24.16)		
Master’s/Ph.D.	85	18.8	96.24(20.17)		
** Income**					
High	48	10.6	102.89(24.28)	One way ANOVA*	*P* = 0.04
Average	268	59.4	96.66(21.78)		*F* = 3.09
Dissatisfied	135	29.9	93.43(24.32)		
** Job status**					
Employed	243	53.9	96.92(24.15)	One way ANOVA	*P* = 0.833
Unemployed	124	27.5	95.97(22.69)		*F* = 0.183
Retired	84	18.6	95.28(19.68)		

*Post hoc analysis show that there is statically significance between single and divorce (p = 0.012) and high and dissatisfied income (p = 0.037).

### Translation Procedure

After the translation and verification process by Robin Cohen, a Persian questionnaire with one phrase about family caregivers’ overall quality of life and 16 items under seven subscales were obtained.

### Face Validity

Face validity was verified using ten family caregivers. The items did not change, while assessing face validity due to their simplicity and clarity.

### Construct Validity

The three methods of EFA, CFA, and convergent validity were employed to assess the construct validity of scale.

#### Exploratory Factor Analysis

A KMO value was found to be 0.832, and Bartlett’s test of sphericity was significant (X^2^ = 2222.856, df = 120, *p* = 0.000). Three factors were extracted and labeled since they described 51.85% of the total variance of family caregivers’ quality of life (Table 2).

**TABLE 2:** Exploratory Factor Analysis of the Persian Version of the QOLLTI – F v3.

Factor	Items	Factor loading%	Variance
Factor 1	Q6	0.869	29.58
	Q7	0.816	
	Q5	0.795	
	Q8	0.707	
	Q11	0.527	
Factor 2	Q13	0.839	13.05
	Q12	0.824	
	Q1	0.571	
	Q9	0.532	
	Q10	0.474	
	Q2	0.473	
Factor 3	Q14	0.725	9.21
	Q15	0.713	
	Q16	0.645	
	Q4	0.567	
	Q3	0.539	
Cumulative%			51.85

#### Confirmatory Factor Analysis

**FIGURE 1:**
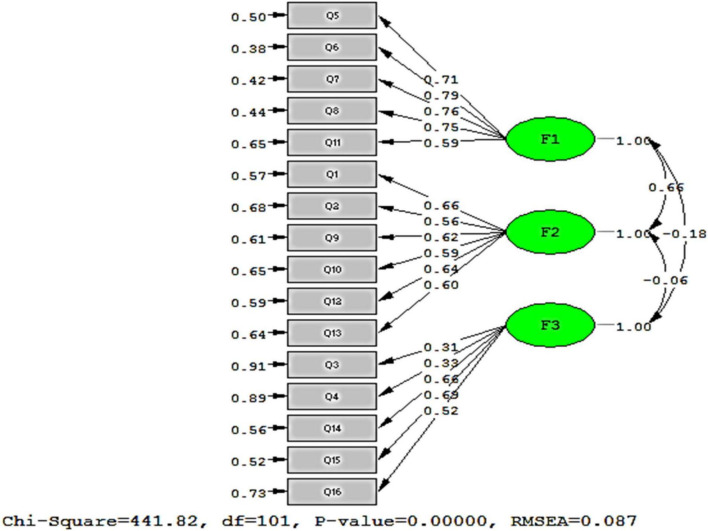
The final structure of the model.

In the CFA, the model showed a good fit. The indices examined for goodness fit included: NFI = 0.98, RMSEA = 0.087, GFI = 0.89, standardized root mean SRMR = 0.070, CFI = 0.91, and IFI = 0.91. The results of the CFA are provided in Figure 1

#### Convergent Validity

For the convergent validity, a significant correlation was found among participants’ total scores of QOLLTI – F v3 and Persian version of ZBI (*r* = –0.196, *P* = 0.000). In other words, the higher the burden of care, the lower the quality of life of the family caregiver will be.

### Reliability

For scale reliability, internal consistency was obtained to be 0.719 using Cronbach’s Alpha Coefficient. Besides, stability was confirmed using the test-retest method. Pearson correlation coefficient was reported to be 0.71.

## Discussion

During COVID-19 pandemic, family caregivers’ quality of life was severely affected. Family caregivers provide measures associated with the symptom management, treatment, and the patient’s physical, psychological, and social care (Arias-Rojas et al., 2021). Therefore, addressing caregivers’ problems is essential to provide appropriate treatment and social support to patients and ensure the continuity of care (Karimirad et al., 2018). Consequently, QOLLTI-F is a valid tool to assess family caregivers’ quality of life. The present study was conducted to investigate the psychometric properties of the Persian version of the QOLLTI – F.

This study discreetly performed the translation process until a final Iranian version was obtained. Similar to the results of this study, the face validity of the original English version of scale was assessed by Cohen et al. with a sample size equal to 30 caregivers (Cohen et al., 2006). In addition, face validity was assessed in the German version (Schur et al., 2014) by 30 caregivers, the Latin American–Spanish version (Arias-Rojas et al., 2021) by 21 caregivers, the Swedish version (Axelsson et al., 2020) by 15 patients and nine caregivers, and in the Malaysian version (Alnjadat et al., 2014) by 10 caregivers, which indicated that the items were straightforward and clear after being translated.

The present study used EFA, CFA, and convergent validation to confirm the construct validity using the ZBI. Regarding the number of extracted factors (3 factors) following EFA, the present study is consistent with Arias-Rojas et al.’s study (Arias-Rojas et al., 2021), conducted to prepare the American–Spanish version. However, the result of EFA in this study was inconsistent with the study by Schur et al. (2014) to develop the German version (4 factors extracted), the study by Alnjadat et al. (2014) in preparing the Malaysian version (7 factors extracted), and the study by Cohen et al. (2006) to prepare the original version of the scale (7 factors extracted). This difference is probably due to the disease nature in the studied populations and sample size. In contrast to the present study, in the studies by Alnjadat et al. (2014) and Schur et al. (2014), no CFA was performed; however, the researchers in this field recommend performing this stage of construct validity in future studies.

Pearson correlation between two scales (QOLLTI-F-V3 scale and the ZBI) indicated an appropriate correlation and confirmed the convergence validity of this scale. In other words, the higher the burden of care, the lower family caregivers’ quality of life will be.

Similar to the present study, in the study by Schur et al. (2014) the Integrative Hope Scale (IHS) was used to assess concurrent validity. Besides, the Hospital Anxiety and Depression Scale (HADS) and the Subjective Carers Burden questionnaire were used to assess discriminant validity, revealing a significant relationship between the two scales.

The present study indicated the appropriate reliability (Cronbach’s alpha and Test-retest) of the scale. This result was in line with the results of the original version of the QOLLTI-F scale with reliability (Cronbach’s alpha = 0.86) and stability (Test-retest = 50–0.79) (Cohen et al., 2006). Furthermore, the results of the present study regarding reliability were in agreement with the results of the study conducted by Schur et al. (2014) to localize the German version (Cronbach’s alpha = 0.83) and stability (Test-retest = 0.92) (Schur et al., 2014), the study by Alnjadat et al. (2014) to localize the Malaysian version (Cronbach’s alpha ≥ 0.74), and the study by Arias-Rojas et al. (2021) to localize the Latin American–Spanish version of QOLLTI-F scale (Cronbach’s alpha = 0.83) and stability (Test-retest = 0.87).

To conclude, according to the psychometric properties of tool in the Persian version, it can be stated that it is an excellent scale to be used to measure family caregivers’ quality of life. Another advantage of the tool is its conciseness, which requires ten minutes to complete. This tool was widely validated, and its different versions are available in various languages.

### Limitations

So far, an adequate number of tools in the field of palliative care in Iran have not been made or psychometric. As a result, one of our constraints was the absence of psychometric methods to assess the study’s criterion validity. Another disadvantage was that this research only included family caregivers of COVID-19 patients. There are other types of life-threatening illnesses, such as cancer, advanced heart failure, chronic obstructive pulmonary disease, in which the quality of life may be affected dissimilarly. The researchers recommend that this study be performed on other patients who use palliative care services. In addition, future studies are needed to investigate this new structure using other tests to evaluate concurrent and discriminant validity and fully validate the QOLLTI-F in the Persian language.

## Conclusion

QOLLTI – F is a valid and reliable questionnaire to measure the family caregiver’s quality of life during a life-threatening illness. Therefore, it can be used in clinical evaluation and research to improve family caregivers’ quality of life in Iranian society. This tool can be used in various diseases and medical centers, including hospitals, nursing homes, and hospice centers.

## Data Availability Statement

The original contributions presented in the study are included in the article/supplementary material, further inquiries can be directed to the corresponding author.

## Author Contributions

AF: conceptualization, methodology, draft preparation, and data collection. AE and MR: methodology and draft preparation. SH: methodology and draft preparation. MM: supervision, data collection and draft preparation. AK, MJ, BA, and MSR: data collection and draft preparation. SB: supervision, conceptualization, methodology, draft preparation and data collection. All authors contributed to the article and approved the submitted version.

## Conflict of Interest

The authors declare that the research was conducted in the absence of any commercial or financial relationships that could be construed as a potential conflict of interest.

## Publisher’s Note

All claims expressed in this article are solely those of the authors and do not necessarily represent those of their affiliated organizations, or those of the publisher, the editors and the reviewers. Any product that may be evaluated in this article, or claim that may be made by its manufacturer, is not guaranteed or endorsed by the publisher.
